# Comment on the “YOPRO-1: A Cyanine-Based Molecular Rotor Probe for Amyloid Fibril Detection”

**DOI:** 10.1021/acsabm.5c00784

**Published:** 2025-06-06

**Authors:** Karina Kwapiszewska

**Affiliations:** Institute of Physical Chemistry, Polish Academy of Sciences, Kasprzaka 44/52, Warsaw 01-224, Poland

The detection of amyloid fibrils in neurodegenerative diseases remains a noteworthy issue in both research and clinical settings. The identification of YOPRO-1 as a potential amyloid fibril sensor offering superior sensitivity and selectivity appears promising.^1^ The authors of the original work^1^ presented a remarkable YOPRO-1 fluorescence enhancement upon binding to insulin fibrils in both buffer and biological fluids (serum). Although these results suggest excellent potential for neurodegenerative disease diagnosis, several crucial limitations associated with YOPRO-1’s cellular interactions demand careful consideration before endorsing its application for amyloid detection, especially in complex biological environments.

In recent work, it has been demonstrated that YOPRO-1 penetrates living human cells.^2^ This property, while potentially advantageous for specific applications,^3,4^ introduces significant complications for amyloid detection. Cellular uptake means that YOPRO-1 will not exclusively interact with extracellular amyloid deposits but will bind to various intracellular components. This nonspecific distribution compromises the probe’s ability to provide accurate spatial information about amyloid localization, particularly in tissue samples containing both cells and extracellular matrix.

A further consequence of YOPRO-1 cellular uptake is its high fluorescence in cells (Figure 1). It was demonstrated that YOPRO-1 binds to double-stranded nucleic acids, which include not only nuclear DNA but also rRNA and tRNA, which are abundant in the whole cell volume.^2^ This characteristic directly undermines the probe’s utility in complex biological matrices, precisely the capability highlighted as an advantage in the original study. The high background signal would significantly reduce the signal-to-noise ratio, potentially masking the detection of early amyloid formations and limiting the sensitivity in practical applications.

**Figure 1:**
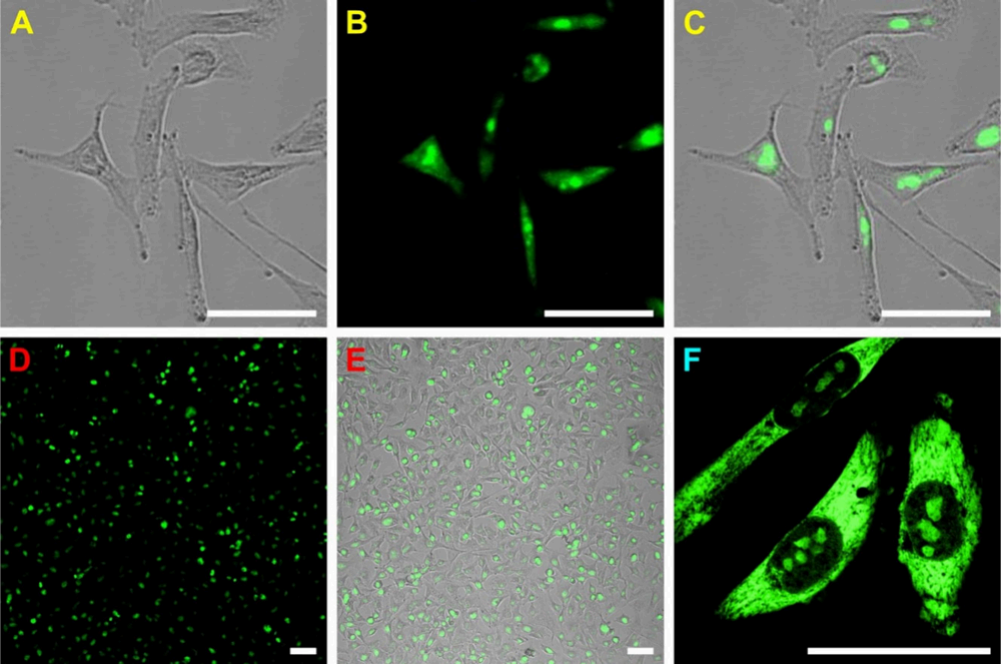
Intracellular localization of YOPRO-1 in HeLa cells. (A–C) Staining viable HeLa cells in culture (A) with 0.5 μM YOPRO-1 yields bright green fluorescence (B and C, merged image). (D, E) The YOPRO-1 staining is not uniform in the population of cells (B, green fluorescence channel; E, merged image). (F) Confocal imaging of fixed HeLa cells treated with 0.2 μM YOPRO-1 reveals subcellular localization, mainly in the cytoplasm and nucleoli, neglecting solely double-stranded DNA intercalation. Scale bars represent 50 μm. Experimental procedures are described in the Supporting Information.

The properties mentioned above and the YOPRO-1 primary use as a DNA intercalator^5^ raise another concern: DNA intercalation suggests potential mutagenic activity, posing safety risks for *in vivo* applications. DNA intercalator dyes can induce mutations *in vivo* by inserting between DNA base pairs, which distorts the DNA helix and interferes with replication and transcription processes.^6^ Additionally, specific intercalating agents have been shown to inhibit topoisomerase II, leading to double-strand breaks and chromosomal rearrangements that contribute to their mutagenic potential.^7^ The mutagenic properties of these compounds explain why many DNA intercalators are used as chemotherapeutic agents against cancer cells while presenting concerns for laboratory safety when handling these dyes. Comprehensive toxicological studies should evaluate these risks before any *in vivo* application or even extensive *ex vivo* use with patient samples.

The original study demonstrates YOPRO-1’s functionality in human serum, which is a cell-free environment.^1^ This may be a promising and exciting direction for early diagnosis of amyloid fibers in peripheral blood. However, the original text does not explicitly highlight this direction as being the most promising one. Instead, references to the necessity of crossing the blood-brain barrier and the frequent mention of imaging studies suggest a preference for *in vivo* applications of YOPRO-1. This approach, however, may entail significant risks and limitations, as discussed above. Nevertheless, the study of Shahane et al. provides a valuable and innovative contribution to the field, opening new perspectives for the diagnostic use of fluorescent probes in neurodegenerative diseases. While YOPRO-1 demonstrates promising properties for amyloid detection in simplified systems, its cellular penetration, DNA intercalation, and RNA binding characteristics present significant limitations that may restrict its practical utility and safety. Moreover, its blood-brain barrier penetration—stated in the original study—was not confirmed and may be another obstacle.^8^ These limitations should be thoroughly addressed before YOPRO-1 can be recommended as a reliable tool for amyloid detection in complex biological environments or considered for any clinical diagnostic applications.

## Supplementary Material


