# Supplementary material for: Comment on the “YOPRO-1: A Cyanine-Based Molecular Rotor Probe for Amyloid Fibril Detection”
Source: ACS Appl Bio Mater. 2025 Jun 6;9(1):1–2. doi: 10.1021/acsabm.5c00784 (PMC12776570; doi:10.1021/acsabm.5c00784)
Supplement: Supplementary file 1 [file mt5c00784_si_001.pdf]

Supporting Information

to

Comment on the "YOPRO-1: A Cyanine-Based Molecular Rotor Probe for Amyloid Fibril Detection"

*Karina Kwapiszewska**

Institute of Physical Chemistry, Polish Academy of Sciences, Kasprzaka 44/52, Warsaw, POLAND

* corresponding author: kkwapiszewska@ichf.edu.pl

SI. 1 Materials and methods

Cell culture

HeLa cells were obtained from the American Type Culture Collection (ATCC, Manassas, USA) and maintained as a monolayer under standard laboratory conditions (37 °C, 5% CO2). The cells were cultivated in complete growth medium consisting of Dulbecco's modified Eagle's medium (DMEM) containing 1 g/L glucose (Institute of Immunology and Experimental Technology, Wrocław, Poland), enriched with $10\%_{v/v}$ fetal bovine serum (FBS), $1\%_{v/v}$ L-glutamine (Sigma-Aldrich), and $1\%_{v/v}$ antibiotic solution comprising streptomycin (10 mg/mL) (Merck) and penicillin (10,000 U/mL) (Sigma-Aldrich). Cell passages were performed using 0.25% Trypsin-EDTA solution (Sigma-Aldrich) to release cells from the culture surface.

Living cells observations

HeLa cells were plated in 8 well-chambered cover glass plates (Cellvis) 24 hours before experimental procedures. Subsequently, the growth medium was exchanged with PBS containing calcium and magnesium ions supplemented with 0.5 µM YO-PRO-1, and cells were incubated for 30 minutes. Cellular imaging was performed using a Nikon A1 confocal microscope controlled by NIS Elements software (Nikon). Data acquisition included two channels: a transmitted light detector (TD) to capture quasi-brightfield images showing cellular morphology and a green fluorescence channel (excitation wavelength = 485 nm) to detect YOPRO-1 fluorescence.

Fixed cells imaging

HeLa cells were placed in 8 well-chambered cover glass plates (Cellvis) 24 hours before testing. After this, cells were washed with PBS and then fixed with 4% formaldehyde in PBS for 10 minutes. The fixed cells were rinsed with PBS and made permeable using 0.2% Triton X-100 in PBS for 5 minutes. The permeabilization buffer was removed by washing with fresh PBS, and samples were then ready for staining. YOPRO-1 was used at a final concentration of 0.2 µM. Images were collected using a Nikon A1 confocal microscope through a green fluorescence channel (485 nm excitation wavelength) to observe the intracellular distribution of fluorescent YOPRO-1.
